# Editorial: Next generation *in vitro* models to study chronic pulmonary diseases, volume II

**DOI:** 10.3389/fmed.2025.1683514

**Published:** 2025-09-03

**Authors:** Tony J. F. Guo, Alen Faiz, Emmanuel T. Osei, Simon D. Pouwels

**Affiliations:** ^1^Department of Biology, University of British Columbia, Kelowna, BC, Canada; ^2^Centre for Heart Lung Innovation, St. Paul's Hospital, Vancouver, BC, Canada; ^3^Faculty of Science, Respiratory Bioinformatics and Molecular Biology, University of Technology Sydney, Sydney, NSW, Australia; ^4^Department of Pulmonary Diseases, University Medical Center Groningen, Groningen, Netherlands; ^5^Department of Pathology and Medical Biology, University Medical Center Groningen, Groningen, Netherlands; ^6^GRIAC Research Institute, University of Groningen, Groningen, Netherlands

**Keywords:** 3D *in vitro* models, 2D and 3D co-culture models, 3D mechanical alveolar organoids, Air–liquid Interface (ALI) 3D *in vitro* model, senolytic therapy in COPD models, pneumothorax risk factors

Chronic respiratory diseases, such as asthma and chronic obstructive pulmonary disease (COPD), are leading causes of morbidity and mortality worldwide, and pose a significant burden on healthcare systems (1–3). Improving our understanding of their pathophysiologies is crucial for the development of better therapeutics, with progress in this area increasingly reliant on data derived from *in vitro* experiments and models (4).

*In vitro* cell culture models provide a reliable and accessible method for conducting scientific experiments without using live animals or humans (4, 5). In recent years, these models have rapidly advanced, progressing from static and rigid 2D cell culture systems to advanced 3D models that incorporate features such as soft extracellular matrices, fluid flow, stretch, and co-culture of several different cell types (5–8). These innovations have enhanced the physiological relevance of *in vitro* systems, making their findings more translatable to *in vivo* biology.

A notable advancement in the field is the development of an affordable, customizable open-source alternative to commercial microfluidic models (9). This large airway-on-chip model has a cell culture area comparable to that of a 24-well plate, enabling the collection of sufficient sample volumes for assays such as multi-omics analyses. The model consists of two large cell culture chambers with independent medium or air flow, separated by a semi-permeable membrane, and supports up to 2 × 10^4^ adherent structural lung cells per chamber. It allows for the close contact co-culture of various lung cell types, including airway epithelial cells, fibroblasts, smooth muscle cells, and endothelial cells. Epithelial cells can be cultured at an air–liquid interface (ALI) to promote differentiation into a pseudostratified epithelium and to allow exposure to airborne particles. Requiring only a syringe or peristaltic pump and a 3D printer, this open-source model offers a practical entry point into advanced 3D lung cell culture.

Another significant recent advancement is the development of a complex, 3D, vascularized tri-culture airway model (6). In this system, a bio-printed hydrogel composed of 80% polyethylene glycol diacrylate (PEGDA) and 20% gelatin methacrylate (GelMa) was embedded with lung fibroblasts, while epithelial and endothelial cells were cultured on the apical and luminal sides of the hydrogel, respectively. This vascularized, bio-printed, tri-culture model offers a more physiologically relevant representation of the human airway and is adaptable, allowing for the incorporation of other cell types to increase complexity.

**Figure 1:**
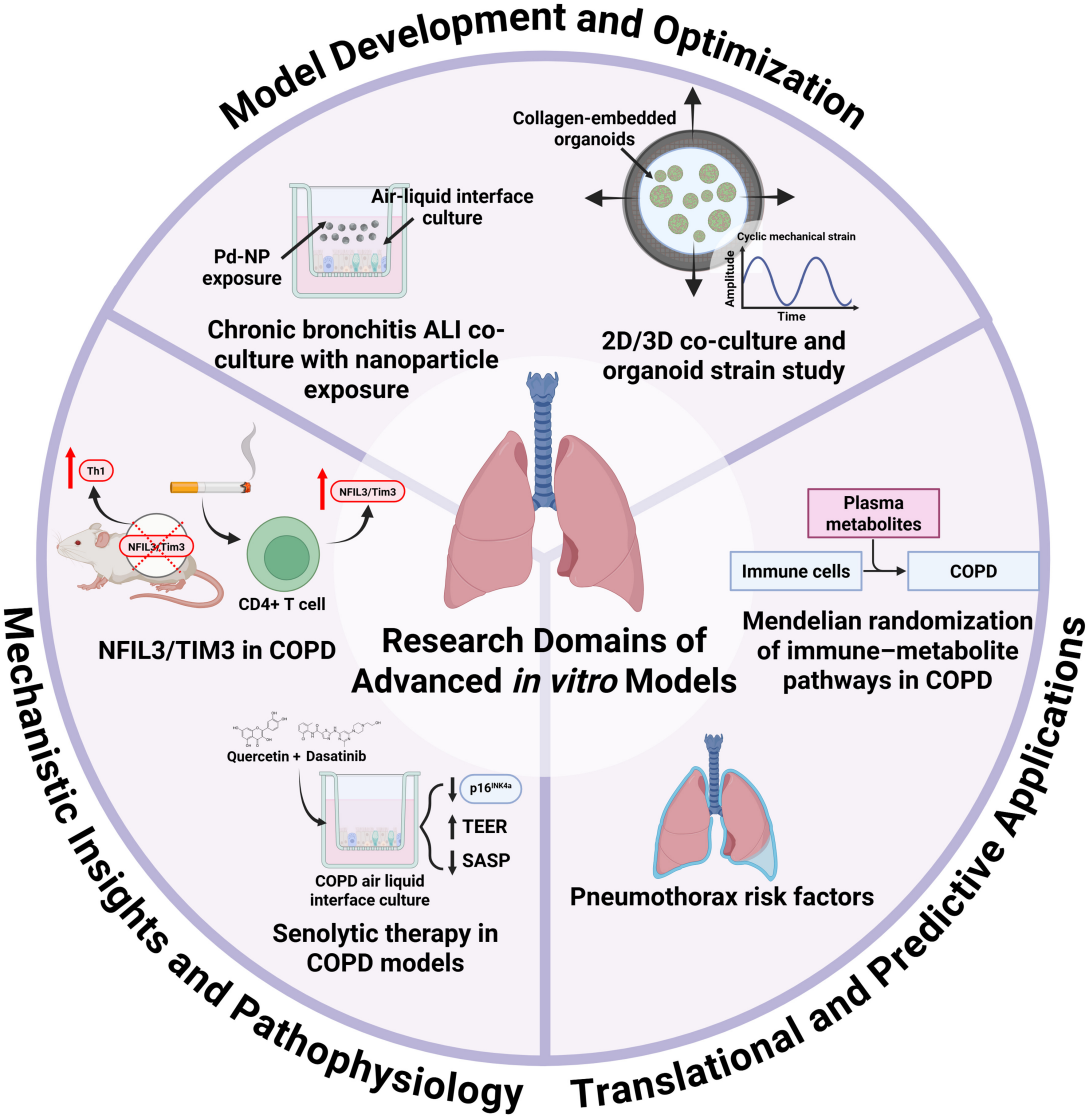
Research domains of advanced *in vitro* models for the study of chronic pulmonary diseases. This Research Topic includes six articles that collectively highlight the diverse research applications of advanced *in vitro* respiratory models. The development and optimization of these systems is critical, as demonstrated by Ji et al., who showcased the utility of air–liquid interface cultures for studying metal nanoparticle exposure, and by Al Yazeedi et al., who characterized multiple models, including alveolar organoids, under mechanical strain. *In vitro* platforms are also powerful tools for elucidating disease mechanisms and pathophysiology: Ke et al. reported that cigarette smoke exposure upregulates NFIL3/Tim3, validating this in a knockout model that revealed Th1 imbalance; Baker et al. showed that COPD epithelial cells maintain a senescent phenotype in air–liquid interface culture, with senolytic treatment reducing senescence markers. Furthermore, advanced models can bridge clinical and epidemiological findings with translational and predictive applications. For example, they could be used to study cellular and matrix responses to injury in pneumothorax, as suggested by the retrospective risk-factor analysis by Ding et al., or to explore biochemical responses and pathways, as identified by Cao et al., whose Mendelian randomization analysis linked immune cell phenotypes to COPD via plasma metabolites.

In the Research Topic, titled “*Next Generation In Vitro Models to Study Chronic Pulmonary Diseases—Volume II*”, six articles highlighted the latest developments in respiratory *in vitro* models (Figure 1). The first study introduced a novel chronic bronchitis ALI co-culture model comprising 16HBE bronchial epithelial cells and MRC-5 lung fibroblasts, optimized for the exposure to aerosolized palladium nanoparticles (Pd-NP), a component of vehicle exhaust (Ji et al.). To induce a chronic bronchitis phenotype, the cells were stimulated basolaterally with recombinant human interleukin-13 throughout a 2-week ALI differentiation period. Nanoparticle exposure was performed using the XposeALI^®^ system. The study developed both chronic and non-chronic bronchitis cell-line ALI models and reported PD-NP-induced inflammatory responses, oxidative stress, and altered markers associated with tissue injury and repair.

The second study in this Research Topic presented the characterization of 2D and 3D co-cultures, along with an alveolar epithelial-fibroblast organoid model, to investigate the effects of mechanical strain (Al Yazeedi et al.). A549 epithelial cells and MRC-5 fibroblasts were co-cultured and subjected to cyclic mechanical strain using a Flexcell stretching bioreactor. Using the collagen I-embedded organoid model, the authors demonstrated that 24 h of cyclic strain with amplitudes and frequencies that mimic pathological breathing patterns altered cellular morphology, proliferation, inflammation and cytotoxicity. These dynamic multicellular models provide a valuable platform for investigating strain-induced inflammatory and fibrotic responses relevant to lung diseases.

Next, ALI-cultured primary bronchial epithelial cells from COPD patients and a cigarette smoke–exposed murine model were used to investigate the effects of senolytic therapy on markers of senescence (Baker et al.). The study reported that the senescent phenotype of COPD-derived epithelial cells was maintained following ALI differentiation. Treatment with a combination of the senolytics dasatinib and quercetin reduced H_2_O_2_-induced senescence *in vitro*. In the cigarette smoke-exposed murine model, the same senolytic cocktail treatment reduced markers of senescence and alveolar inflammatory cell burden. These findings suggest that senolytic therapy may offer a strategy to reduce senescence-associated inflammation in COPD.

Another study in this Research Topic investigated the role of the NFIL3/TIM3 pathway in Th1 imbalance in COPD (Ke et al.). Using *Havcr2* (encoding TIM3) and *Nfil3* knockout mice that were exposed to cigarette smoke for 24 weeks to induce emphysema, along with isolated CD4^+^ T cells, the authors demonstrated that disruption of this pathway increased Th1 cell infiltration, elevated IFN-γ expression, and caused a more severe emphysematous phenotype. These findings suggest that Tim3 expression in CD4^+^ T cells is regulated by NFIL3 and that TIM3 has anti-inflammatory effects, counteracting cigarette smoke-induced inflammation and providing protection against the development of emphysema. Advanced co-culture models incorporating T cells, epithelial cells, and stromal cells could help elucidate the mechanisms of TIM3 signaling and how its modulation may influence tissue remodeling.

This Research Topic also included a retrospective analysis that aimed to identify risk factors for pneumothorax following ^125^I particle implantation in patients with advanced lung cancer (Ding et al.). A needle-pleura angle of less than 50°, pre-operative CT evidence of emphysema, the presence of atelectasis, and lesions in the left lung fissure were identified as independent risk factors. Advanced 3D models that simulate alveolar structures and pleural layers could help assess cellular and matrix responses to mechanical injury by replicating needle puncture and particle implantation.

Finally, a study used Mendelian randomization and publicly available genome-wide association study data to explore the causal relationships among immune cells, COPD and metabolic mediators (Cao et al.). The analysis identified eight immune cell phenotypes influenced by eight specific metabolites, which may serve as biomarkers for the early detection of COPD.

This Research Topic highlights the value of advanced *in vitro* models in respiratory research (Figure 1). These physiologically relevant systems support the investigation of disease mechanisms, environmental exposures, and therapeutic responses. They also offer a practical way to build on insights from patient data and *in vivo* studies. As they evolve, they are well-positioned to bridge the gap between basic and translational research.
